# Cellular and Molecular Connections between Autophagy and Inflammation

**DOI:** 10.1155/2015/398483

**Published:** 2015-06-29

**Authors:** Pierre Lapaquette, Jean Guzzo, Lionel Bretillon, Marie-Agnès Bringer

**Affiliations:** ^1^Université Bourgogne Franche-Comté, UMR PAM, Équipe Vin, Aliment, Microbiologie, Stress, 21000 Dijon, France; ^2^Agrosup Dijon, UMR PAM, Équipe Vin, Aliment, Microbiologie, Stress, 21000 Dijon, France; ^3^INRA, UMR1324 Centre des Sciences du Goût et de l'Alimentation, 21000 Dijon, France; ^4^CNRS, UMR6265 Centre des Sciences du Goût et de l'Alimentation, 21000 Dijon, France; ^5^Université Bourgogne Franche-Comté, Centre des Sciences du Goût et de l'Alimentation, 21000 Dijon, France

## Abstract

Autophagy is an intracellular catabolic pathway essential for the recycling of proteins and larger substrates such as aggregates, apoptotic corpses, or long-lived and superfluous organelles whose accumulation could be toxic for cells. Because of its unique feature to engulf part of cytoplasm in double-membrane cup-shaped structures, which further fuses with lysosomes, autophagy is also involved in the elimination of host cell invaders and takes an active part of the innate and adaptive immune response. Its pivotal role in maintenance of the inflammatory balance makes dysfunctions of the autophagy process having important pathological consequences. Indeed, defects in autophagy are associated with a wide range of human diseases including metabolic disorders (diabetes and obesity), inflammatory bowel disease (IBD), and cancer. In this review, we will focus on interrelations that exist between inflammation and autophagy. We will discuss in particular how mediators of inflammation can regulate autophagy activity and, conversely, how autophagy shapes the inflammatory response. Impact of genetic polymorphisms in autophagy-related gene on inflammatory bowel disease will be also discussed.

## 1. Introduction

Autophagy is an evolutionarily conserved process. Constitutive autophagy is required for cellular housekeeping (e.g., elimination of damaged or long-lived organelles) [1]. It is a highly sensitive process that cells are induced in response to a wide range of stressful conditions (physical, chemical, or metabolic) in order to maintain cellular homeostasis [2]. While the inflammatory responses are generally beneficial for host protection, this process needs to be spatially and temporally tightly regulated to avoid a state of excessive and/or sustained inflammation that is potentially detrimental. Indeed, prolonged exposure of tissues and organs to high concentration of inflammatory mediators represents a stressful environment for cells and can result in severe damage [3]. Since an abnormal inflammation could disrupt cellular homeostasis, it is not, thus, surprising that autophagy contributes to damp inflammatory responses. Autophagy acts by at least two means to protect cells from excessive long lasting inflammation: (i) indirectly by allowing efficient clearance of damaged organelles (mitochondria, e.g.,) or intracellular pathogenic microorganisms that both constitute potent inflammatory stimuli and (ii) directly by suppressing proinflammatory complexes. Naturally, regulatory networks that control autophagy activity are able to sense output signals from various inflammatory mediators-associated signaling, allowing a proper modulation of the process according to inflammation state. In this review, following a brief introduction on molecular mechanisms controlling autophagy, we will make an overview of interrelations existing between inflammation and autophagy. Facing tremendous number of studies describing relationships between inflammatory mediators and autophagy, it is nearly impossible to be completely exhaustive, but we will highlight some of the best-characterized interactions between these two processes. In the last part of the review, we will discuss in more detail the crosstalk between autophagy and inflammation during pathophysiological situations, especially inflammatory bowel diseases.

## 2. Autophagy: How Does It Work?

### 2.1. Different Types of Autophagy

Three main forms of autophagy have been described in mammalians. Macroautophagy corresponds to the sequestration of cytoplasmic structures into double- or multimembrane vesicles termed autophagosomes. Complete autophagosomes then transit along microtubules to deliver their content to degradative compartments, lysosomes, forming autolysosomes [1]. The term microautophagy refers to the direct engulfment of the cytosolic material by invagination of the lysosomal membrane [4]. The third form of autophagy is chaperone-mediated autophagy (CMA), during which, proteins containing a pentapeptide motif (KFERQ-like sequence), are recognized by the cytosolic chaperone hsc70 (heat shock cognate protein of 70 kDa) and its cochaperones that deliver them to the surface of lysosomes. The substrate-chaperone complex binds to the lysosomal protein LAMP-2A (lysosome-associated membrane protein type 2A) and the substrate is unfolded. Multimerization of LAMP-2A is required for substrate translocation inside the lysosome [5]. In this review, we will focus only on macroautophagy (hereafter referred to as autophagy) and its interrelations with inflammatory processes.

Autophagy was first described as a nonselective bulk degradation process, sequestering a portion of the cytosol and used by the cell during nutrient deprivation period. In light of studies during last decade, it turns out that autophagy can also be selective, allowing, under certain conditions, the sequestration of specific substrates such as mitochondria (mitophagy), endoplasmic reticulum (ER- or reticulophagy), lipid droplets (lipophagy), peroxisomes (pexophagy), endosomes, lysosomes, secretory granules, ribosomes (ribophagy), cytoplasmic aggregates (aggregaphagy), inflammatory proteins, and invading pathogens (xenophagy) [6]. Structures targeted for destruction by autophagy are often ubiquitinated. A series of autophagy receptors, termed SLRs, for Sequestosome 1- (SQSTM1-) like receptors contain ubiquitin-binding domain (UBD) associated with a LIR (LC3-interacting region) motif and act as adaptors between K48- or K63-linked polyubiquitin chains on a targeted-substrate and ATG8 paralogs (LC3, GABARAP), bridging autophagic cargoes to nascent autophagosomes [6]. Members of SLRs family include p62/SQSTM1, NBR1, NDP52, and optineurin. Substrates can also be delivered to autophagosome in ubiquitin-independent manner, as exemplified by mitophagy. In some cases, autophagy-mediated degradation of mitochondria relies on polyubiquitylation of proteins at the outer mitochondrial membrane and is dependent on PINK1 (PTEN-induced putative kinase protein 1) and the E3 ligase Parkin. In other cases, however, mitophagy is dependent on mitochondrial outer-membrane proteins (e.g., NIX) that can directly link mitochondria to autophagosomal membranes* via* their own LIR domain. Finally, an alternative way has been observed in neuronal cells and involves externalization of an inner mitochondrial membrane phospholipid, named cardiolipin, to the outer mitochondrial membrane and its direct recognition by LC3 [7, 8].

### 2.2. Molecular Machinery of Autophagy

Autophagy can be divided into 6 main steps: initiation, vesicle nucleation, elongation, membrane elongation, closure, maturation, and degradation. Initiation step leads to the formation of an isolation membrane, called phagophore, most often in close vicinity with the endoplasmic reticulum (ER). Various organelles, including the ER, the Golgi apparatus, mitochondria, the plasma membrane, and endosomes, have been proposed to serve as membrane reservoir for phagophore generation and growth. Initiation of autophagy requires two protein kinases complexes: (i) the ULK1/2-ATG13-FIP200 complex, which is coupled with the autophagy suppressor TOR complex 1 (mTORC1), and (ii) the Beclin1-Vps34-Vps15-ATG14 complex. This last complex is usually inhibited by interactions with proteins from the Golgi apparatus, antiapoptotic Bcl2 proteins, and other signals transducers [9]. Given the fact that autophagy is a highly dynamic process, its activation is largely dependent on a set of posttranslational modifications such as phosphorylation, acetylation, and ubiquitylation [10]. The mTORC1 complex consists of the mTOR kinase, which is a master cell-growth regulator integrating numerous intracellular and extracellular signals (growth factor, nutrients, and cellular energy status), G*β*L, PRAS40, and raptor. Under basal condition, the mTORC1 complex associates with the ULK1/2-ATG13-FIP200 complex and phosphorylates ULK1/2 and ATG13, resulting in the inhibition of ULK1/2 kinase activity. Under stressful conditions (e.g., nutrient deprivation), AMPK activates ULK1/2 (in complex with ATG13 and FIP200) directly by site-specific phosphorylation and indirectly by inhibiting mTORC1 [11]. Dephosphorylation of mTOR-dependent inhibitory sites on the ULK1/2-ATG13-FIP200 complex releases ULK1/2 activity allowing autophosphorylation of this complex and its interaction to the ATG101 protein in an ATG13-dependent manner [12]. These events lead to the subsequent recruitment and activation of the Beclin1-ATG14-Vps34-Vps15 complex at the membrane, inducing nascent phagophore formation. This vesicle nucleation step relies on dynamic assembly of both autophagy initiation complexes (ULK1/2-ATG13-ATG101-FIP200 and Beclin1-ATG14-Vps34-Vps15) on the exocyst, a scaffolding protein complex involving the Ras-like small G-protein RalB and its effector Exo84, which acts as an activation platform for core autophagy machinery [13]. The Beclin1-associated phosphatidylinositol 3-kinase class III, Vps34, marks the site where the phagophore emerges from the ER, by generating a PI3P-enriched structure, called omegasome. This event leads to the recruitment of PI3P-binding proteins such as DFCP1 (double FYVE-containing protein 1), Alfy, and WIPI (WD-repeat domain phosphoinositide interacting) family proteins. Members of the WIPI family, WIPI1, WIPI2, and WIPI4, recognize PI3P accumulation at the nascent autophagosome and are necessary for the recruitment of the autophagosome elongation complex ATG12–ATG5–ATG16L1 complex [14].

Elongation of the phagophore membrane involves two ubiquitin-like proteins: ATG12 and ATG8. Similarly to ubiquitination, ATG12 is conjugated to ATG5 (substrate) by ATG7 (E1-like enzyme) protein and ATG10 (E2-like enzyme). Then, the ATG5-ATG12 complex can interact noncovalently with ATG16L1 and associates with the phagophore. The second ubiquitin-like reaction involves ATG7 (E1-like enzyme) and ATG3 (E2-like enzyme) enzymes and is required for conjugation of ubiquitin-like molecules of the ATG8 family (LC3, GABARAP, and GATE-16) to the lipid phosphatidylethanolamine. Whereas ATG5-ATG12-ATG16L1 complex will dissociate from closed autophagosomes, LC3-II, the lipidated form of LC3, remains associated with the autophagosome until fusion with the lysosome [15]. It has been observed that ATG5-ATG12-ATG16L1-positive/LC3-negative pre-autophagosomal structures coalesce through SNARE-mediated homotypic fusions, thereby increasing the size of the membrane constituting the phagophore, a prerequisite for optimal acquisition of LC3 and progression from autophagosome precursor to phagophore [16]. Although its function is not still fully understood, the transmembrane protein ATG9 is also proposed to orchestrate membrane delivery to the phagophore assembly site [17].

Closure of autophagosome is thought to require LC3-conjugation system [18]. However, the lack of mutant cells defective for this step does not allow for precise molecular insights. Once edges of the phagophore are sealed, sequestering substrates to be degraded, the autophagosome migrates along microtubules to a perinuclear location and interacts with endosomes and lysosomes [19]. Fusion of these organelles is orchestrated by SNARE proteins (VAMP3 and VAMP7) and Rab GTPases (at least Rab7 and Rab11) [20–22]. The Beclin1 protein, which is essential for autophagy initiation, also plays a role in autophagosomal maturation by indirectly modulating Rab7 protein activity [22]. The last stage of autophagy is the efflux into the cytosol of metabolites (amino acids, sugars, and lipids) that are generated by autolysosomal degradation. It involves transmembrane proteins termed permeases. In yeast, recycling of amino acids from autophagosomes involves three vacuolar amino acid permeases Atg22, Avt3, and Avt4 [23]. Interestingly, mTOR, which is the master suppressor of autophagy, is reactivated upon autophagy termination by amino acids release and stimulates extrusion of lysosome-derived membranes from autolysosomes to form new functional lysosomes [24].

## 3. Mediators of Inflammation Regulating Autophagy

### 3.1. Innate Immune Receptors

At the cellular level, presence of pathogens is detected by “pattern recognition receptors” (PRRs) located at the plasma membrane (Toll-like receptors (TLRs) 1, 2, 4, 5, and 6), at endosomal membranes (TLR3, TLR7, TLR8, and TLR9) or in the cytosol (Nod-like receptors (NLRs), retinoic acid-inducible gene-I- (RIG-I-) like receptors (RLRs), C-type lectin like receptors (CLRs)) [25–28]. These innate immune receptors recognize highly conserved structural motifs present on microbes termed “pathogen-associated molecular patterns” (PAMPs), such as the bacterial cell wall components lipopolysaccharide (LPS), flagellin, and lipoproteins; bacterial and viral nucleic acids; and the fungal cell wall components zymosan and mannan. They also detect “danger-associated molecular patterns” (DAMPs) that signalize host cellular damage. Connections exist between autophagy and innate immune receptors, where PRRs serve as sensor for microbial presence and autophagy ensures their intracellular elimination through lysosomal degradation. It is important to notice that PRRs repertoire varies substantially from one cell type to another; thus by contrast to starvation-induced autophagy, PRRs-mediated autophagy will be more cell type dependent. In addition, PRRs distribution could be influenced by cell polarity. For example, in the colon, TLR5 is only exposed on the basolateral side of enterocytes and is absent from apical side [29].

Screening of PAMPs library for their effects on autophagy showed that prototype ligands of various TLRs, including TLR1, TLR3, TLR4, TLR5, TLR6, and TLR7, induce autophagy in mouse and human macrophages [30, 31]. TLR9-dependent induction of autophagy has also been reported in human colonic epithelial cells stimulated by bacterial CpG motifs [32]. Invading pathogens, such as* Listeria monocytogenes* (*L. monocytogenes*) and* Staphylococcus aureus*, have also been shown to activate autophagy in macrophages in a TLR2-dependent manner and thereby triggered their own elimination [33, 34]. Interestingly, activation of autophagy through TLR stimulation by agonists enables also the elimination of noncognate intracellular pathogens [30].

Connections between TLR and autophagy are complex to study since various downstream signaling effectors are engaged, according to the innate immune receptor activated. Data discrepancies exist regarding the adaptor (Myd88 versus TRIF) involved in autophagy induction by TLR4 stimulation in macrophages [31, 35]. Delgado et al. showed that activation of autophagy* via* TLR7 upon macrophages stimulation with ssRNA is dependent on MyD88 [30]. Mechanistically, connection between TLR-signaling and autophagy is supposed to be mediated by the adaptor proteins TRIF or Myd88 that are found to coimmunoprecipitate with Beclin1 and reduce the binding of Beclin1 to the inhibitory protein Bcl2, leading to autophagy activation [31]. Physical association of Myd88 with mTOR has also been reported allowing activation of master transcription factors (interferon-regulatory factor- (IRF-) 5 and IRF-7) for proinflammatory cytokine- and type I IFN-genes [36]. In addition, mycobacterial infection of human macrophages and zebrafish embryos induced DRAM1 (DNA damage-regulated autophagy modulator 1) mediated selective autophagy in a Myd88 and NF-*κ*B-dependent manner [37].

Nod receptors stimulation by bacterial peptidoglycan components induces autophagy and results in enhanced bacterial killing and antigen presentation [38, 39]. The molecular interaction between the cytoplasmic Nod1 and Nod2 receptors and autophagy has been nicely shed in light by D. Philpott group. They showed that ATG16L1 interacts with Nod receptor that enables recruitment of autophagy machinery at the bacterial entry site and the subsequent delivery of bacteria into autophagosomes for degradation [39]. “NODophagy” entails other autophagy-related proteins such as ATG5, ATG7, and receptor-interacting serine-threonine kinase-2 (RIPK-2) [38]. Autophagy regulation by Nod proteins is evolutionarily conserved, since the drosophila homologue PGRP-LE, recognizing also peptidoglycan fragments, is able to induce autophagy upon* L. monocytogenes* challenge [40]. Modulation of autophagy by other NLR family members, involved in inflammasomes assembly, has been reported [41–43]. Activation of AIM2- or NLRP3-mediated inflammasomes triggers the activation of the small G protein RalB and autophagosome formation [43]. Through binding to Exo84, RalB induces the assembly of catalytically active ULK1 and Beclin1-Vps34 complexes [13]. Flagellin recognition by the NLR proteins Naip5 and NLRC4 stimulates autophagosome turnover [41].

Conversely, NLRs can negatively regulate autophagy. NLRC4 and NLRP4 negatively regulate autophagic processes through an association with Beclin1. In addition, NLRP4 physically interacts with the C-VPS complex (VPS11, VPS16, VPS18, and Rab7) that controls membrane tethering and fusion of vacuolar membranes, thereby blocking maturation of autophagosomes to autolysosomes [44].

To summarize, PRRs, by sensing various microbial products from bacteria, virus and fungi are able to modulate autophagy at multiple steps (initiation and maturation).

### 3.2. Cytokines

By binding to their specific receptors located at the cytoplasmic membrane, cytokines modulate autophagy. In a general way, Th1 cytokines (IL-2, TNF-*α*, and IFN-*γ*) are considered as autophagy inducers whereas Th2 cytokines (IL-4, IL-5, IL-6, IL-10, and IL-13) and anti-inflammatory cytokines are regarded as autophagy repressors [45]. Cytokines that have been reported to induce autophagy encompass IL-1, IL-2, IL-6, TNF-*α*, TGF-*β*, and IFN-*γ* [45].

Autophagy induction by cytokines may constitute an important mechanism in the elimination of invasive pathogens. IFN-*γ* illustrates this mechanism since it promotes degradation of intracellular* Mycobacterium tuberculosis *and* Chlamydia trachomatis* by inducing autophagy [46, 47]. Induction of autophagy by IFN-*γ* involves immunity-related GTPase such as murine Irgm1, human IRGM, Irga6, and members of the 65-kDa guanylate binding protein family [46–49]. Of note, unlike the* Irgm1 *gene in mice, the human Irgm1 ortholog,* IRGM*, is not responsive to IFN-*γ*, but interestingly it remains able to induce autophagy upon infection in epithelial cells and macrophages [49–51]. A single nucleotide polymorphism (SNP) in* IRGM* (261T) is associated with resistance to* M. tuberculosis* infection in people carrying this protective variant [52]. In* Irgm1−/−* macrophages, an alternative activation pathway of autophagy by IFN-*γ*, depending on the p38 MAPK has been described [53]. In human hepatocellular carcinoma cells, IFN-*γ* induces autophagy through IRF-1 signaling pathway and this autophagy activation is associated with cell death [54]. Induction of autophagy by type I IFNs has also been reported in several cancer cell lines and involved JAK/STAT and PI3K-mTOR pathways [55]. Type I IFN proteins include the IFN-*α* subtypes, IFN-*β* and IFN-*ω*, and play pleiotropic functions, such as antiviral, antiproliferative, and immunomodulatory activities.

The proinflammatory cytokine and death ligand, TNF-*α*, represents also an autophagy inducer in various cell types, including T-lymphoblastic leukaemic cells, osteoclasts, or Ewing sarcoma cells [56–58]. TNF-*α* regulation of autophagy has been shown to be dependent on various signaling pathways including JNK and Erk signaling and via the production of reactive oxygen species (ROS) [59–62]. The role of TNF-*α* in autophagy is also supported by studies on* M. tuberculosis*, since reactivation of tuberculosis associated with anti-TNF-*α* treatments (infliximab, adalimumab, certolizumab pegol, and etanercept) is suspected to, at least partially, depend on suppression of the antibacterial autophagic process [63, 64].

Various effects on autophagy have been reported for IL-6 depending on considered tissue. IL-6 enhanced autophagic activity in myeloid cells and mouse pancreatic tumor cells [65, 66]. In contrast, IL-6 overexpression blocks autophagy in human bronchial epithelial cells by supporting interaction between Beclin1 and the antiapoptotic Bcl2 protein Mcl-1 [67]. Other cytokines that have been described to induce autophagy include IL-1*β*, which is suspected to be an endogenous inhibitor of the mTOR pathway [68] and TGF-*β*, which stimulates autophagy through various signaling pathways such as JNK, Smad, and TAK1 pathways [69, 70].

Conversely, some Th2 and anti-inflammatory cytokines exert autophagy-suppressive functions. Regarding IL-4 and IL-13 cytokines, they inhibit starvation-induced autophagy through stimulation of PI3K/Akt signaling pathway and are able to counteract IFN-*γ*-induced autophagy in a signal transducer and activator of transcription 6 (STAT6) dependent manner [71]. The anti-inflammatory cytokine IL-10 has also been described to inhibit starvation-induced autophagy by stimulating the PI3K/Akt signaling pathway [72].

### 3.3. Reactive Oxygen Species

Several lines of evidence indicate that reactive oxygen species (ROS) are early autophagy inducers upon nutrient deprivation. This is supported by data showing that treatment with antioxidants partially or completely reverses the process [73]. Under nutrient deprivation, it has been reported as the expulsion of reduced glutathiones (GSH), which are powerful antioxidants, by the plasma membrane translocator ABCC1MRP1 (multidrug resistance protein 1) in the extracellular milieu [74]. This results in a shift of the intracellular redox state toward oxidizing conditions and may favor oxidation of redox-sensitive proteins, inducing consequently the early autophagy. Mitochondria represent the principal source of ROS that triggers autophagy. It is postulated that superoxides and hydrogen peroxide, the main ROS produced by mitochondria upon nutrient deprivation, could activate autophagy by at least two ways. One molecular mechanism that has been described is the activation of the AMPK (5′-adenosine monophosphate-activated protein kinase), an energy sensor of the cell, through S-glutathionylation of its cysteines. S-glutathionylation is a posttranslational modification of cysteines frequently induced in cells as a response to ROS production. AMPK stimulates autophagy by phosphorylating TSC2 (tuberous sclerosis complex 2), an inhibitor of mTORC1, raptor, a component of the mTORC1 complex, and by phospho-activating ULK1 [11, 75, 76]. Another mechanism by which ROS induce autophagy is by oxidizing a cysteine residue near the catalytic site of the cysteine protease ATG4, thereby stimulating its proteolytic activity and enhancing autophagy [77, 78].

### 3.4. Inflammation-Related Transcription Factors

Until recently, transcription control of autophagy was clearly underappreciated, but compelling evidences during last years demonstrate that a network of transcription factors is involved in fine-tuning of autophagy. Some transcription factors (TFs), which orchestrate inflammatory responses, have been also described as transcriptional regulators of the core autophagy genes. A prototypical example of these TFs is the nuclear factor-*κ*B (NF-*κ*B) p65/RelA family member, which has been shown to upregulate* BECN1* and* SQSTM1* transcription [79, 80]. Other inflammation-related TFs that are able to modulate autophagy genes transcription include hypoxia inducible factor-1 (HIF-1), JUN, signal transducers, and activators of transcription (STAT) 1 and STAT3 [81]. HIF-1 stimulates autophagy by enhancing transcription of BNIP3 (BCL2/adenovirus E1B 19 kDa protein-interacting protein 3) and BNIP3L (BNIP3-like) encoding genes [82]. These two proteins, which belong to the BH3-only Bcl2 family proteins, are known to activate autophagy. C-JUN is recruited to the* Beclin1 *and* ATG8* genes promoter regions in response to ceramide treatment and thereby enhances transcription of these genes [83, 84]. A role as negative autophagy regulators has been described for STAT1 and STAT3, since an impaired expression of those TFs correlates with an increased autophagy activity and stimulates transcription of some* Atg* genes including* Atg12* and* Beclin1* [85, 86]. TFs belonging to the nuclear receptors family, such as peroxisome proliferator-activated receptor *α* (PPAR*α*), PPAR*γ*, and Farnesoid X-activated receptor (FXR), which function as ligand-activated TFs and modulate inflammatory response, can also modulate autophagy [87, 88]. PPAR*α* and FXR have opposite effect on autophagic genes transcription, since pharmacological activation of PPAR*α* leads to an upregulation of the expression of autophagy genes, whereas FXR activation leads to a repression. Copetti and colleagues demonstrate that these two nuclear receptors compete for binding to the same site on autophagy genes promoters [79]. Inflammatory state has also been described to modulate the activity of transcription factor EB (TFEB), a master regulator that orchestrates the expression of autophagy and lysosomal genes [89]. Hence, TFs represent an additional complex layer of regulation; thereby inflammation deeply affects autophagy-associated transcriptional program.

## 4. Regulation of Inflammation by Autophagy

### 4.1. Regulation of Inflammasomes

Inflammasomes are intracellular signaling platforms that detect a set of substances emerging during infections, tissue damage, or metabolic imbalances and that proteolytically activate the highly proinflammatory cytokines IL-1*β* and IL-18 [90]. These multimeric protein complexes usually consist of three partners: an inflammasome sensor protein, which can be a PAMP- or DAMP-detecting module in the form of a NLR, such as NLRP3 and NLRC4, or an endogenous DNA (released from mitochondria) detecting module such as AIM2 (absent in melanoma 2), the adaptor protein ASC and caspase-1 that enzymatically processes pro-IL-1*β* and pro-IL-18 for their activation. Activation of inflammasomes also causes pyroptosis, which corresponds to a rapid and proinflammatory form of cell death [90].

Autophagy acts as a negative regulator of inflammasomes. Mice those are deficient in ATG16L1, which is essential for autophagy, present higher IL-1*β* and IL-18 levels in sera in response to LPS stimulation or during colitis [91]. These results have been confirmed by pharmacological (3-methyladenine treatment) or genetical (LC3B and Beclin1) inhibitions of autophagy that all result in higher IL1-*β* production upon PAMPs stimulation [43, 92]. At the opposite, TLR- or rapamycin-induced autophagy leads to reduced amount of pro-IL1-*β* [93]. However, the molecular mechanisms by which autophagy regulates inflammasome activation are not yet fully understood. Autophagy could act directly by sequestering and degrading ubiquitinated-inflammasomes and pro-IL-1*β* molecules or indirectly by lowering endogenous sources inducing inflammasome formation, such as mitochondrial ROS and DNA [43, 92–94].

### 4.2. p62/SQSTM1 as a Regulator of the Oxidative Stress Response

In addition to its role in clearance of polyubiquitinated proteins and bacteria by autophagy [95, 96] and in the regulation of the nutrient-sensing pathway [97], p62/SQTM1 is also involved in the regulation of inflammatory mediators production. The p62/SQTM1 protein can induce the upregulation of oxidative stress response genes, particularly those controlled by Nrf2, an antioxidant transcription factor. Under basal condition, Nfr2 is sequestered in the cytoplasm by Keap1. Upon oxidative stress, p62/SQSTM1 interacts and competes with the Nrf2-binding site on Keap1, leading to the stabilization and the nuclear translocation of Nrf2 and transcription of target genes, including key ROS scavengers encoding genes [98]. Thus, the Nrf2 activation is reinforced by degradation of p62/SQSTM1-Keap1 complex by autophagy and through the activation of p62/SQSTM1 transcription by Nrf2.

### 4.3. Secretion of Mediators of Inflammation

Besides the conventional secretion process by which proteins endowed with a leader peptide undergo modification in the ER, transit through the Golgi apparatus, and are secreted upon fusion of post-Golgi carriers with the plasma membrane, an unconventional secretion process based on autophagy has been described. This secretion process first described in yeast is involved in the secretion of proteins devoid of leader peptide. It is present in mammalian cells and termed “secretory autophagy” (also referred to as “autosecretion” [99] or “type III secretion” [100]). The principal features defining secretory autophagy are the involvement of ATG proteins and autophagy process, and dependence on Golgi reassembly and stacking protein, GRASP55 and GRASP65. The secretory autophagy is a rising investigation area and a number of points remain to be elucidated to understand how autophagy orients peptide to secretion or simple degradation. It has been reported in mammalian cells that secretory autophagy contributes to the secretion of proinflammatory cytokines (IL-1*β* and IL-18) and alarmins (high mobility group box (HMGB)-1), under transient and specific circumstances [101]. This process depends on ATG5, inflammasome, GRASP55, and Rab8a.

### 4.4. Control of Macrophages Polarization

Mononuclear phagocytes and among them macrophages play a central role in the orchestration and expression of innate and adaptive immune responses. One of the hallmarks of these cells is their high diversity and plasticity. In tissues, in response to a large variety of environmental cues (e.g., growth factors, cytokines, microbial products, or glucocorticoids), mononuclear phagocytes undergo transcriptional reprograming that shape their phenotype and functions, M1 (classical) and M2 (alternative) phenotypes, being the extremities of a continuum of activation states [102]. Macrophage differentiation from myeloid lineage relies on sustained expression of the transcription factor PU.1. Then, depending on the macrophage microenvironment, M1 macrophage polarization is mediated by STAT1 and IRF-5, whereas M2 polarization is supported by STAT6, IRF-4, and PPAR*γ* [103]. Multiple other signaling molecules, transcription factors, epigenetic mechanisms, and posttranscriptional regulators involved in fine-tuning of macrophage polarization have been characterized [104]. Of note, macrophage polarization has been shown to be a transient and reversible state [105]. Schematically, M1 macrophages stimulate a proinflammatory response against intracellular microorganisms and tumors cells, whereas M2 macrophages are immunosuppressive cells and participate in cancer progression by supporting key processes such as angiogenesis, tissue repair, and remodeling.

In addition to playing role in macrophages migration and differentiation, which will not be discussed in this review, evidences showed that autophagy is involved in the control of macrophage polarization. Binding of hepatoma-derived factors to TLR2 stimulate M2 macrophage polarization, a phenotype expressed by most of tumor-associated macrophages (TAM) from established tumors, by regulating cytoplasmic NF-*κ*B level through selective autophagy [106, 107]. Pharmacological treatment with bafilomycin A1, a lysosomal inhibitor, or genetic defects (knockdown of* ATG5*) that impaired autophagy prevents degradation of NF-*κ*B (p65) and forces the M2 macrophages to secrete high levels of inflammatory cytokines which characterized M1 phenotype. In addition, the mTOR pathway has been described as a critical element of the monocyte differentiation to TAM. Inhibition of mTOR through rapamycin treatment caused the monocytes to differentiate toward a M1 phenotype, whereas activation of mTOR by RNA interference-mediated knockdown of the mTOR repressor TSC2 induced the differentiation of monocytes toward a M2 macrophage phenotype [108]. A defect in CatS, a cathepsin whose expression is upregulated in several tumors, is associated with accumulation of autophagosomes and attenuation of the autophagic flux in macrophages. It has been recently demonstrated that Cat S-mediated autophagic flux is required to maintain polarization of the M2 TAMs phenotype [109].

## 5. Autophagy in Human Inflammatory Diseases, the Example of Crohn's Disease

Given the multiple roles played by autophagy in immune homeostasis, malfunctions of this process have been associated with the pathogenesis of a variety of diseases. Hence, autophagy may impact on the onset or progression of various human diseases associated with a chronic inflammatory state, including inflammatory bowel diseases (IBDs), Paget's disease, infectious diseases (tuberculosis), lysosomal storage disorders, and autoimmune disorders (systemic lupus erythematosus, rheumatoid arthritis, multiple sclerosis, and type 1 diabetes) [110]. Among them, IBDs are the most studied inflammatory diseases for their link with autophagy alteration and especially Crohn's disease (CD). CD and ulcerative colitis (UC) represent the two major forms of idiopathic IBD. It is now widely accepted that the etiology of IBD involves environmental and genetic factors that lead to dysfunction of the epithelial barrier with consequent deregulation of the mucosal immune system and responses to gut microbiota [111]. Human genetic studies over the last decade and experimental studies have revealed the keystone role played by autophagy in the disorders associated with CD.

### 5.1. Autophagy Genes as Risk Factors for Crohn's Disease

The initial identification of the link between autophagy and CD arose from genome-wide association studies (GWAS), which revealed the association between a coding single nucleotide polymorphism (SNP) in the* ATG16L1* gene of the autophagy core machinery and an increase risk of CD onset [112]. Two others genes associated with autophagy have been confirmed as CD susceptibility genes:* Irgm *(immunity-related GTPase family M) and* LRRK2* (leucine-rich repeat kinase 2) [113, 114]. More recently, whole exome sequencing of CD patients has identified a CD-associated missense variant (V248A) in the autophagy receptor NDP52 [115]. CD-associated polymorphisms in ATG16L1 and IRGM encoding genes have been reliably found in many patient cohorts as reported by a meta-analysis [116, 117]. Of note,* Irgm* polymorphisms are also associated with an increased risk in developing UC, what is not true for other autophagy-related genes associated with CD-susceptibility [116].

Regarding* Irgm*, a synonymous variant within the coding region (rs10065172, CTG > TTG, leucine) was initially linked to CD [114], and this silent polymorphism was found to be in perfect linkage disequilibrium with a 20 kb deletion upstream of* Irgm *[118]. Results showed that the 20 kb deletion does not impair, in a reliable manner, expression of the IRGM mRNA, but that silent polymorphism alters the recognition of the IRGM mRNA by a family of microRNA (miR), miR-196a and miR-196b [51, 118]. In a specific study to identify rare SNPs in autophagy-related genes, a CD-associated SNP in the* Ulk1* gene locus has been identified, but the functional relevance of this genetic variation in the disease is still unknown [119]. Association between granulomas, one of the microscopic hallmarks of CD, and variants in autophagy* ATG4A*,* ATG2A*,* Fnbp1l,* and* ATG4D* genes has also been reported [120]. In addition, three mutations in* Nod2*, including a frameshift mutation (L1007fsinsC) that results in a truncated NOD2 protein and two amino acid substitutions (R702W and G908R), have been reported to be strongly associated with CD onset [121, 122]. Mutations in* Nod2* encoding gene impaired ability of this innate immune receptor to interact with ATG16L1 and recruit the autophagy machinery for degradation of invasive bacteria [39]. ATG16L1, independently of its role in autophagy, acts as a negative regulator of the Nod1 and Nod2-mediated proinflammatory signaling pathways, by negatively regulating the activation of the Rip2 kinase [123]. These studies strengthen the connection existing between* Nod2*, the first gene historically associated with CD etiology, and autophagy-related-genes associated recently with CD by GWAS, highlighting the importance of autophagy in CD onset.

### 5.2. Endoplasmic Reticulum Stress Related Inflammation

ER stress is caused by the accumulation of unfolded or misfolded proteins in the ER. Cells secreting large amounts of protein, such as Paneth cells and Goblet cells in the intestine, are highly susceptible to ER stress for survival and to executing their secretory functions. The unfolded protein response (UPR) enables the cell to resolve the ER stress by facilitating the folding, export, and degradation of proteins accumulating in the ER during stressed conditions. Three ER membrane resident proteins, IRE1 (inositol requiring transmembrane kinase endonuclease 1), PERK (pancreatic ER kinase), and ATF6 (activated transcription factor 6) sense the presence of unfolded protein in the ER lumen and induce a transcriptional program necessary for the UPR. In the absence of misfolded proteins in the ER lumen, IRE1, PERK, and ATF6 are maintained as inactive complexes through association with Grp78 [124].

Complex connections exist between ER stress and inflammation. In mice, genetic deletion of molecules involved in the UPR, such as IRE1*β*, the transactivator of UPR's Xbp1 (X-box binding protein 1) and Agr2 (Anterior gradient 2) target genes, which is a member of the ER protein disulfide isomerase (PDI) gene family are associated with either spontaneous intestinal inflammation and/or increased sensitivity to the experimental induction of colitis [125–127].

In human, genetic studies have identified the UPR-related genes* Xbp1* and* ORMDL3* (orosomucoid-like 3), which is involved in ER calcium homeostasis, as risk loci associated with Crohn's disease [113]. Evidences for increased ER stress response in patients with CD include an increased expression of Grp78 and an increased* Xbp1* splicing in the small intestine and colon of CD patients comparatively to controls [125, 128, 129].

Intestinal epithelial cell-specific deletion of* Xpb1* (*Xbp*1^ΔIEC^) and most notably in Paneth cells leads to autophagy induction linked to ER stress related PERK-eIF2*α* signaling pathway [130]. Interestingly, spontaneous ileitis observed in *Xbp*1^ΔIEC^ mice is converted into severe CD-like transmural ileitis when both mechanisms, ER stress and autophagy, are compromised (*Atg7*/*Xbp*1^ΔIEC^ and* Atg16L1*/*Xbp*1^ΔIEC^ double transgenic mice) [130]. In addition, ATG16L1-dependent autophagy restrains IRE1*α*-mediated NF-*κ*B activation. Thus, autophagy may function as a buffer mechanism in intestinal epithelial cells protecting cells from inflammation generated upon sustained ER stress.

Evidences showed that the crosstalk between UPR and autophagy may be bidirectional. Defective autophagy promotes ER stress in hepatocytes [131] and increased* Xbp1* splicing and Grp78 expression have been observed in *Atg*16*L*1^ΔIEC^ mice crypt compartment compared to wild-type [130]. In addition, patients with CD carrying the *ATG*16*L*1^T300A^ risk variant frequently exhibit ER stress in their Paneth cells, in contrast to those harboring the normal variant [132].

### 5.3. Inflammation Related to Impaired Xenophagy

The term “xenophagy” refers to the control of intracellular pathogens by autophagy. Invasive bacteria that escape into the cytosol such as* Shigella flexneri* or* Listeria monocytogenes* or those that reside in intracellular vacuoles such as* Salmonella* Typhimurium or* Mycobacterium tuberculosis* can be entrapped into autophagosomes and delivered to lysosomes for degradation. Key molecules that target bacteria for xenophagic degradation had been identified and include NLRs (NOD1 and NOD2), ubiquitin, galectins, and diacylglycerol (DAG) [96]. Recent evidences show that cell infection with pathogens, such as* S. flexneri* or* S.* Typhimurium, triggers an amino acid starvation response, an inhibition of the mTOR signaling, and thereby induces autophagy [133]. This suggests that xenophagy response against invasive pathogens might derive from primordial metabolic stress response, reinforcing the link existing between cell metabolism and immune cellular defense.

Dysbiosis of the fecal-, lumenal-, and mucosal-associated microbiota have been described in CD patients [134] and substantial evidence supports an abnormal colonization of the mucosa and lesions of patients with ileal form of CD by* Escherichia coli *belonging to the pathovar designated adherent-invasive* E. coli* (AIEC) [135, 136]. One putative consequence of impaired autophagy is an uncontrolled pathobionts overgrowth due to defective intracellular bacterial clearance. “Pathobiont” term refers to microorganisms that are usually recognized as harmless but that can become pathogens under certain environmental conditions (e.g., host detrimental diet or host genetic susceptibility). Such a hypothesis is supported by data demonstrating that inflamed tissues of the terminal ileum of CD patients with the risk allele of* ATG16L1* harbor a higher abundance of three pathosymbiont groups, Enterobacteriaceae (mostly* E. coli*), Bacteroidaceae (mostly* Bacteroides fragilis* group), and Fusobacteriaceae, in comparison with those with the protective allele [137]. It has been shown that autophagy protects against dissemination of pathobionts and true invasive intestinal pathogens, since intestinal epithelial cell-specific deletion of* Atg5* exhibits increased dissemination of these bacteria to extraintestinal sites [138]. Silencing expression of* ATG16L1* by siRNA in human macrophages and epithelial cells and expression of the risk variant of* ATG16L1* by epithelial cells impair AIEC handling by autophagy and favor their persistence within host cells [139, 140]. Monocytes from CD patients homozygous for the* ATG16L1* risk allele showed impaired killing of AIEC under inflammatory conditions compared with those homozygous for the* ATG16L1* protective allele [137]. Similarly impaired xenophagy against AIEC bacteria has been described in dendritic cells isolated from donors with CD-associated Nod2 variants and in peritoneal macrophages isolated from Nod2 knockout mice [38, 139]. Conversely, pharmacological and physiological induction of autophagy overcomes defects in intracellular AIEC clearance. Similarly to ATG16L1 and Nod2, decreased expression level of IRGM totally impairs autophagy initiation and leads to uncontrolled replication of AIEC bacteria. An unexpected result was that overexpression of IRGM, as observed in the intestinal mucosa of CD patients (compared to controls), also altered the autophagosome maturation step, supporting intracellular AIEC persistence [51].

Risk polymorphisms associated to CD in the autophagy-related* Nod2*,* ATG16L1,* and* Irgm* genes are associated with aberrant inflammatory responses. Defects in autophagy caused by alteration of the expression of autophagy-related genes lead to an exacerbated inflammatory response in human THP-1 macrophages [139]. Surprisingly, in a mouse model of* ATG16L1* deficiency, this abnormal higher inflammatory response has been shown to confer protection against uropathogenic* E. coli *(UPEC) infection, by allowing an accelerated clearance of these bacteria in an IL-1*β*-dependent manner [141, 142]. At the opposite, forced induction of autophagy decreased proinflammatory cytokine release. Recently, it has been shown that AIEC infection upregulated levels of microRNAs like miR-30c and miR-130a in intestinal epithelial cells by activating NF-*κ*B [143]. Upregulation of these microRNAs inhibited autophagy by reducing the levels of ATG5 and ATG16L1 and led to increased numbers of intracellular AIEC and exacerbated inflammatory response.* In vitro*, blocking of miR-30c and miR-130a in AIEC-infected cells restored functional autophagy. Interestingly, ileal samples from patients with CD have increased levels of these same microRNAs and reduced levels of ATG5 and ATG16L1 [143].

## 6. Conclusion

**Figure 1:**
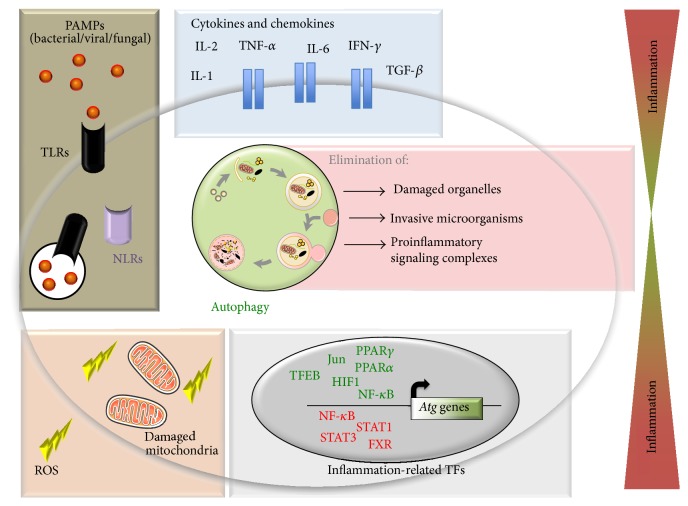
Autophagy and inflammation. By integrating output signals from PRRs (brown box), cytokines, and chemokines (blue box) and ROS (orange box), autophagy regulatory network is able to dynamically respond to inflammation. Transcriptionally, inflammation-related transcription factors shape transcriptional program of autophagy (grey box). Active autophagy reduces inflammation at least by mediating damaged organelles clearance, lowering microorganisms intracellular load and degrading inflammatory mediators (red box).

As illustrated by studies detailed above in this review, autophagy and autophagy-related proteins are essential components modulating inflammatory response either directly by acting on stability or secretion of inflammatory mediators or indirectly by suppressing intracellular stressors (e.g., damaged organelles, intracellular pathogenic microorganisms, and ER stress). Autophagy regulatory network integrates a wide range of signals from innate immune receptors sensing PAMPs (TLRs and NLRs), cytokines, and ROS and thereby is able to respond appropriately according to the degree of inflammatory state (Figure 1).

Autophagy has a significant impact on health, since it has been shown that specific induction of autophagy can increase lifespan in multiple animal species [144]. We could assume that the ability of autophagy to restrain detrimental side effects of inflammation might contribute to its positive role on health. An interesting parallel, supporting this hypothesis, is the fact that, during aging, there is a decrease in autophagy efficiency [145], concomitantly to an increase in the basal inflammation level [146]. Novel therapies designed to enhance autophagy might represent an attractive strategy to overcome autophagy insufficiencies associated with some human inflammatory diseases, such as CD. Beyond the well-known autophagy inducer, rapamycin, already used in clinic as an immunosuppressive and antitumor drug [147], high-throughput screening studies have identified additional molecules, already FDA approved, that are able to modulate the autophagic process [148, 149]. As a proof of concept,* in vivo* delivery in mice of a specific autophagy inducer peptide, Tat-Beclin1, has demonstrated positive results in the treatment of neurodegenerative disease and viral infections [150]. However, considering the multiple roles of autophagy, further investigations are needed to examine in which situations stimulated autophagy is beneficial and does not generate detrimental side effects.
